# 2366. Impact of the Febrile Podcast and Learning Resource as an Infectious Diseases Education Platform

**DOI:** 10.1093/ofid/ofac492.173

**Published:** 2022-12-15

**Authors:** Sara W Dong, Wendy Stead

**Affiliations:** Beth Israel Deaconess Medical Center & Boston Children's Hospital, Boston, Massachusetts; Beth Israel Deaconess Medical Center, Boston, Massachusetts

## Abstract

**Background:**

Febrile is a digital infectious diseases (ID) learning platform with the aim of providing high quality and accessible ID content. We describe utilization of Febrile as a resource for learning and teaching ID, learner satisfaction, and the perceived impact on clinical practice.

**Methods:**

Febrile was launched in December 2020 as a trainee-focused resource as shown in Figure 1. Content is also promoted on social media. Podcast analytics are available from the media host, Captivate, which is Interactive Advertising Bureau certified. Website and Twitter engagement was obtained from Google and Twitter Analytics, respectively. An online anonymous survey was conducted to assess educational impact using Likert scales and open answer questions.

**Results:**

As of April 26 2022, Febrile has produced 39 episodes and has been downloaded > 107,000 times in all 50 United States (US) and 140 countries. Majority of the audience (62%) listens from within the US, see Fig. 2 for geographic distribution. The website has received > 60,000 views, and the Twitter account has garnered > 8,100 followers.

A total of 224 participants from 30 countries and 37 US states completed the survey, of whom 78 (35%) were ID fellows in training and 71 (32%) were ID faculty physicians. Breakdown of other respondents is in Fig. 3.

Most had listened to either ≥ 21 episodes (57, 25%) or 11–20 episodes (86, 38%). In addition, 72% reported visiting the website and 82% had seen a Febrile infographic. There was a high level of satisfaction with the resources, and 83% of respondents have already recommended Febrile to a colleague (Fig. 3).

Enhancing core ID knowledge was the primary driver for listening among other reasons noted in Fig. 4. Two-thirds of respondents indicated that information they learned from Febrile has changed their practice, and 50% have used Febrile as a way to teach others (Fig. 4). Of note, for those considering ID as a career, Febrile had a favorable impact on perception of the specialty (30/34, 88%). Many respondents also indicated Febrile as a source of community (50%, Fig. 4 and 5).

**Conclusion:**

Febrile is a novel and impactful tool for ID medical education. In addition to providing educational resources, this innovative digital platform holds the potential for meaningful community building and attracting trainees to the field of ID.

**Disclosures:**

**All Authors**: No reported disclosures.

**Figure 1:**
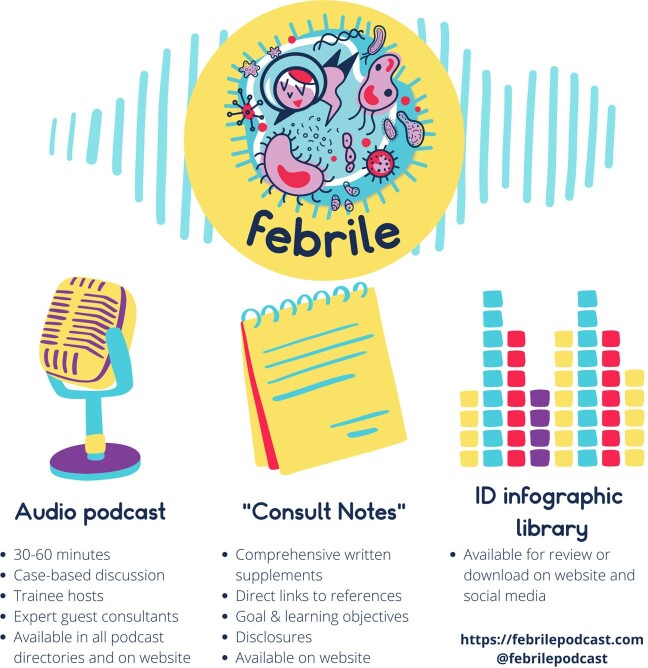
Components of the Febrile Learning Platform

**Figure 2:**
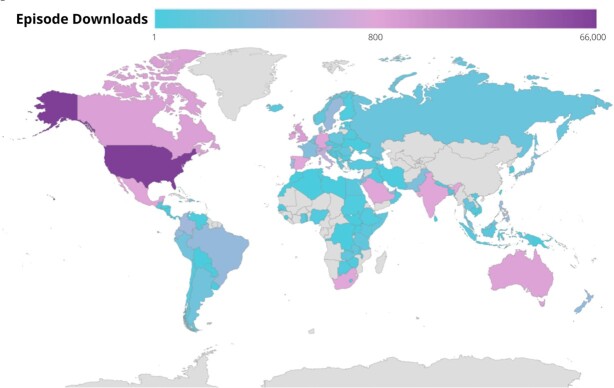
Global Distribution of Febrile Listeners

**Figure 3:**
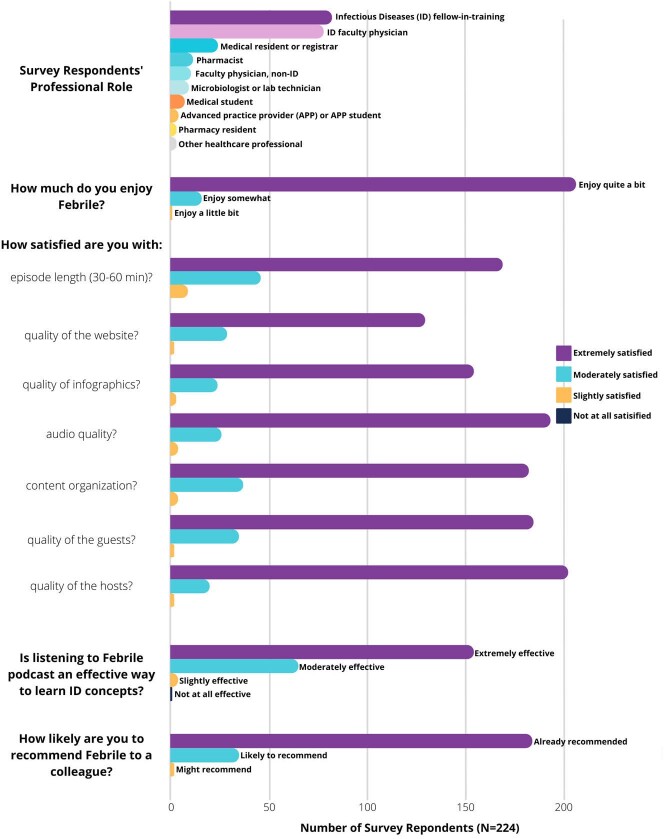
Survey Respondents' Professional Role and Satisfaction with Febrile Resources

**Figure 4:**
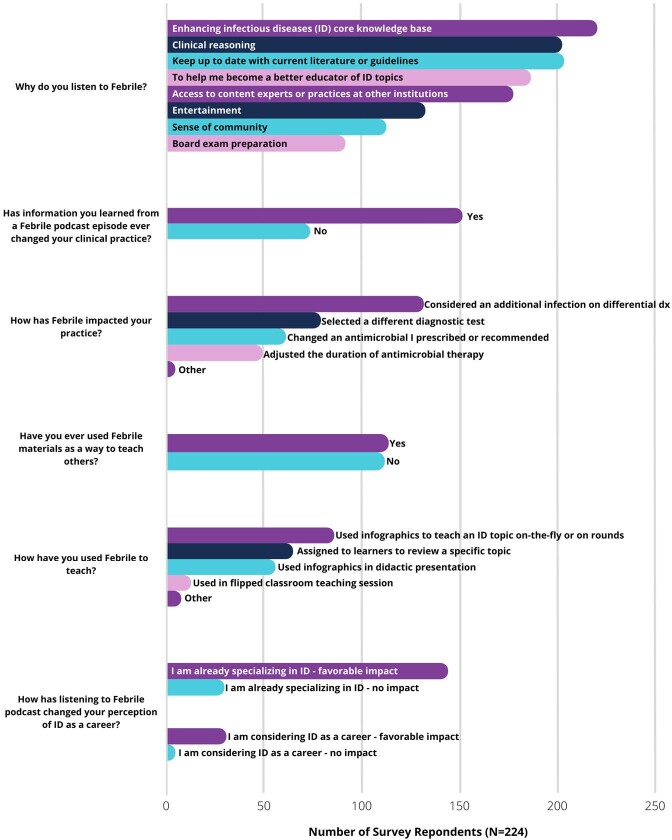
Perceived Impact of the Febrile Learning Platform

**Figure 5:**
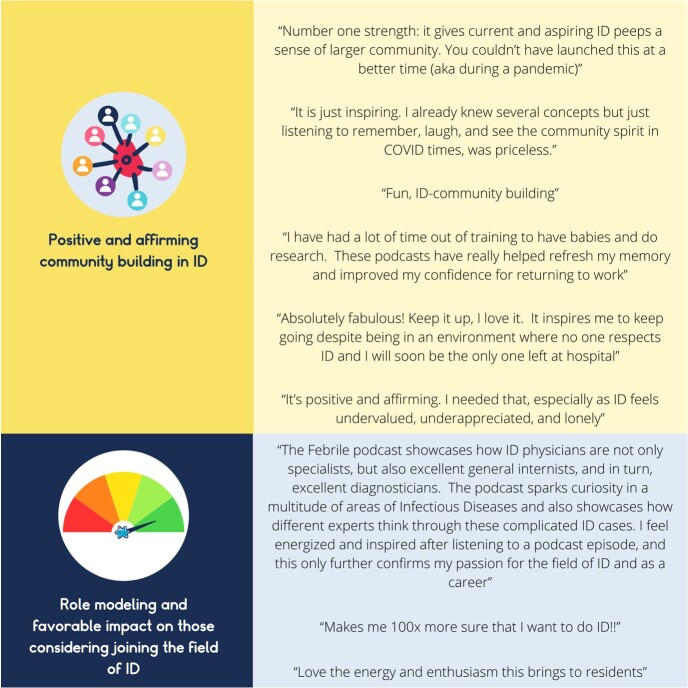
Selection of Respondent Comments

